# Reduction of stearoyl-CoA desaturase (SCD) contributes muscle atrophy through the excess endoplasmic reticulum stress in chronic kidney disease

**DOI:** 10.3164/jcbn.20-24

**Published:** 2020-06-09

**Authors:** Yuki Niida, Masashi Masuda, Yuichiro Adachi, Aika Yoshizawa, Hirokazu Ohminami, Yuki Mori, Kohta Ohnishi, Hisami Yamanaka-Okumura, Takayuki Uchida, Takeshi Nikawa, Hironori Yamamoto, Makoto Miyazaki, Yutaka Taketani

**Affiliations:** 1 Department of Clinical Nutrition and Food Management, Institute of Biomedical Sciences, Tokushima University Graduate School, 3-18-15 Kuramoto-cho, Tokushima 770-8503, Japan; 2 Department of Nutritional Physiology, Institute of Biomedical Sciences, Tokushima University Graduate School, 3-18-15 Kuramoto-cho, Tokushima 770-8503, Japan; 3 Department of Health and Nutrition, Faculty of Human Life, Jin-ai University, 3-1-1 Ohde-cho, Fukui 915-8586, Japan; 4 Department of Nephrology, Faculty of Medical Sciences, University of Fukui, Fukui 910-1193, Japan; 5 Division of Renal Diseases and Hypertension, Department of Medicine, University of Colorado Denver, Aurora, Colorado 80045, USA

**Keywords:** skeletal muscle atrophy, endoplasmic reticulum stress, chronic kidney disease, stearoyl-CoA desaturase, saturated fatty acid

## Abstract

Skeletal muscle atrophy is associated with mortality and poor prognosis in patients with chronic kidney disease (CKD). However, underlying mechanism by which CKD causes muscle atrophy has not been completely understood. The quality of lipids (lipoquality), which is defined as the functional features of diverse lipid species, has recently been recognized as the pathology of various diseases. In this study, we investigated the roles of the stearoyl-CoA desaturase (SCD), which catalyzes the conversion of saturated fatty acids into monounsaturated fatty acids, in skeletal muscle on muscle atrophy in CKD model animals. In comparison to control rats, CKD rats decreased the SCD activity and its gene expression in atrophic gastrocnemius muscle. Next, oleic acid blocked the reduction of the thickness of C2C12 myotubes and the increase of the endoplasmic reticulum stress induced by SCD inhibitor. Furthermore, endoplasmic reticulum stress inhibitor ameliorated CKD-induced muscle atrophy (the weakness of grip strength and the decrease of muscle fiber size of gastrocnemius muscle) in mice and the reduction of the thickness of C2C12 myotubes by SCD inhibitor. These results suggest that the repression of SCD activity causes muscle atrophy through excessive endoplasmic reticulum stress in CKD.

## Introduction

Chronic kidney disease (CKD) is associated with a premature ageing-like phenotype including increased prevalence of cardiovascular disease, osteoporosis, and skeletal muscle atrophy.^(1,2)^ Muscle atrophy is one of the frequent complications in patients with CKD, and is related with CKD prognosis and mortality.^(3)^ In CKD pathogenesis, the activation of proteolytic pathways such as the ubiquitin proteasome system (UPS) and caspase-3 can be primary mechanism of skeletal muscle atrophy.^(2)^

Recent findings suggest that endoplasmic reticulum (ER) stress can also mediate skeletal muscle atrophy.^(4)^ ER stress is caused by disturbances in the structure and function of the ER with the accumulation of misfolded proteins. In response to ER stress, the unfolded protein response (UPR) is initiated by the activation of three molecules: PKR-like ER kinase (PERK), activating transcription factor 6 (ATF6), inositol-requiring enzyme-1 (IRE1).^(5)^ Activated IRE1 releases its endonuclease activity, resulting in splicing of X-box binding protein-1 (*XBP-1*) mRNA, which encodes a transcription factor that can drive transcription of genes, such as *GRP78/BiP*. Activation of the PERK pathway leads to the phosphorylation of an α-subunit of the eukaryotic initiation factor-2α (eIF2α), which promotes the translation of the ATF4 protein. Then, ATF4 induces the C/EBP homology protein (CHOP), which promotes ER stress-induced apoptosis through the activation of caspase-3. Interestingly, the activation of UPR signals in skeletal muscle confirmed in various muscle atrophy models.^(5)^ It has also been reported that ATF4 is a mediator of starvation-induced muscle atrophy.^(6,7)^ Furthermore, the mRNA levels of *ATF4* and *CHOP* are increased in skeletal muscle in hemodialysis patients.^(8)^

Ectopic accumulation of excess lipids, called lipotoxicity, plays a central role in the pathogenesis of cardio-metabolic diseases.^(9)^ On the other hand, the quality of lipids (lipoquality), which is defined as the functional features of diverse lipid species, has recently been a focus of study in the pathology of metabolic diseases. Indeed, some reports indicate that saturated fatty acids (SFAs) and monounsaturated fatty acids (MUFAs) have distinct effects on the peripheral organs by each lipotoxicity. SFAs such as palmitic acid (16:0) and stearic acid (18:0) induce apoptosis, oxidative stress, and ER stress in a variety of mammalian cell lines, whereas MUFAs such as oleic acid (18:1) have no or minimal lipotoxic properties.^(10,11)^ Furthermore, co-treatment with MUFA and SFA inhibits SFA-mediated some effects such as inflammation, ER stress, and apoptosis.^(12,13)^ Intracellular SFAs and MUFAs balance is appropriately controlled by a lipogenic enzyme stearoyl-CoA desaturase (SCD) which catalyzes the conversion of SFAs to MUFAs, mainly 16:0 into palmitoleate (16:1n-7), and stearic acid (18:0) into oleate (18:1n-9).^(14)^ It has been reported that stearic acid and SCD inhibitor (SCDi) induce vascular calcification through ATF4 activation in vascular smooth muscle cells, which is inhibited by oleate.^(15)^ In addition, Woodworth-Hobbs *et al.*^(16)^ recently indicated that the treatment of myotube cells with palmitic acid increases ER stress and caspase-3. Also, palmitic acid dose-dependently lead to a decrease in number, width, and length of myotubes.^(17)^ Interestingly, CKD patients had higher levels of serum stearic acid but not oleic acid, as well as a consequent lower SCD activity compared with subjects with normal kidney function.^(15)^ However, it has not been clarifies that the roles of imbalance between SFAs and MUFAs in ER stress and muscle atrophy in CKD.

In this study, we examined whether (i) CKD shows the repression of SCD activity in skeletal muscle, (ii) co-treatment with oleic acid improves muscle atrophy and ER stress by SCDi, and (iii) ER stress is involved in muscle atrophy in CKD.

## Materials and Methods

### Animal and experimental design

The animal work took place in Division for Animal Research and Genetic Engineering Support Center for Advanced Medical Sciences, Institute of Biomedical Sciences, Tokushima University Graduate School. Eight-week-old male Wister rats and male C57BL/6J mice were purchased from Japan SLC (Shizuoka, Japan) and individually caged in a climate-controlled room (22 ± 2°C) with a 12 h light-dark cycle. Rats were divided into two groups. Control group rats were given a modified-AIN93G diet (Oriental Yeast, Osaka, Japan), and CKD group rat were given a modified-AIN93G diet containing 0.3% adenine (Wako, Osaka, Japan) for 6 weeks. Mice were divided into three groups. Control group mice were given a modifed-AIN93G diet, and CKD group mice were given a modified-AIN93G diet containing 0.2% adenine with or without 4-PBA (Cayman Chemical, Ann Arbor, MI) for 6 weeks. All animals were sacrificed using 0.1 mg/kg body weight of buprenorphine hydrochloride and 50 mg/kg body weight of pentobarbital sodium salt. Composition of the diet is shown in Table 1. The present study was approved by the Animal Experimentation Committee of Tokushima University School of Medicine (animal ethical clearance No. T28-24) and was carried out in accordance with guidelines for the Animal Care and use Committee of Tokushima University School of Medicine.

### Biochemical analysis

Concentrations of plasma creatinine, phosphorus, calcium, and 1,25(OH)_2_D were determined as described previously.^(18)^ Concentrations of blood urea nitrogen (BUN) were determined by urease-GLDH method (Oriental Yeast).

### Muscle histology

Directly after sacrifice, the gastrocnemius muscle (GM) was isolated and immediately frozen in liquid nitrogen cooled acetone. Muscle was embedded in OCT compound and sections (8 µm thickness) were cut with a Leica CM1850 cryostat (Leica). Sections were stained with hematoxylin and eosin (H&E). GM sections were digitally captured using bright field with BZ-II Analyzer (Keyence, Osaka, Japan). The cross-sectional areas of myofibers was measured using the imaging software ImageJ (NIH, Bethesda, MD).

### Fatty acid composition

Fatty acid composition was determined as described previously.^(19)^ Whole lipid was extracted from snap-frozen tissue samples with water/chloroform/methanol (0.7:1:1, v/v/v) containing butylated hydroxytoluene as an antioxidant, according to the method of Bligh and Dyer. The extracted lipids were trans-methylated with HCL-methanol at 100°C for 2 h. The fatty acid methylated esters were separated using gas-liquid chromatography (GC-18A; Shimadzu, Kyoto, Japan) with a capillary column (SP2330; Supelco, Bellefonte, PA). Individual fatty acids were identified by comparing the retention time of each peak with those of the internal standards.

### Grip strength test

Before sacrifice, the grip strength for forelimb was measured by Grip Strength Meter (GPM-101B; Melquest, Toyama, Japan). As a mouse grasped the bar, the peak pull force in newton (N) was recorded on a digital force transducer. Measurements were repeated three times, and the maximum tension from the three measurements was used in the analyses.

### Real-time quantitative PCR analysis

Total RNA was isolated from GM and C2C12 cells using an QIAzol^®^ Lysis Reagent (QIAGEN). Real-time quantitative PCR assays were performed by using an Applied Biosystems StepOne qPCR instrument. In brief, the cDNA was synthesized from 1 µg of total RNA using a reverse transcriptase kit (Invitrogen, Carlsbad, CA) with an oligo-dT primer. After cDNA synthesis, quantitative real-time PCR was performed in 5 µl of SYBR Green PCR master mix using a real time PCR system (Applied Biosystems). The primer sequences used for real-time PCR analysis are shown in Table 2. The quantification of given genes was expressed as the mRNA level normalized to a ribosomal housekeeping gene (β-actin or 18S) using the ΔΔCt method.

### Cell culture and treatment

C2C12 myoblastic cells were cultured as described previously.^(20)^ Briefly, C2C12 myoblastic cells were cultured in Dulbecco’s modified Eagles medium (DMEM; Sigma, St. Louis, MO) containing 10% fetal bovine serum (FBS; Sigma), 100 units/ml penicillin and 100 µg/ml streptomycin at 37°C with 5% CO_2_. At 100% confluence, C2C12 myoblastic cells were fused by shifting the medium to DMEM containing 2% horse serum (HS; Moregate Biotech, Bulimba, Australia). Cells were maintained in 2% HS/DMEM (differentiation medium) for 5 days prior to experiments. Differentiated C2C12 cells were treated with 1 µM CAY10566 (Cayman Chemical) as SCD inhibitor (SCDi) or dimethyl sulfoxide (DMSO) as vehicle control, and 300 µM oleic acid (18:1; Sigma) or free fatty acid-BSA complex as vehicle control. The oleic acid-BSA complex was generated as previously descrived.^(10)^

### Measurement of C2C12 myotube diameters

 Myotube diameters was determined as previously reported.^(21)^ We took 5 photos per cell-culture well at the high-power field on a fluorescence microscopy BIOREVO BZ-9000 (Keyence). We measured the diameter at the middle portion of the myotube with the built-in software BZ-II analyzer. We measured the diameters of 100 myotubes/group.

### Statistical analysis

Data were collected from more than 2 independent experiments and were reported as means ± SEM. Statistical analysis was performed using unpaired *t* test for two-group comparisons or one-way ANOVA with Tukey-Kramer post-hoc test for multiple comparisons. All data analysis was performed using GraphPad Prism 5 software (Graphpad Software, San Diego, CA). *P*<0.05 was considered to indicate statistical significance.

## Results

### Adenine-induced CKD model rats decline SCD activity GM

**Fig. 1:**
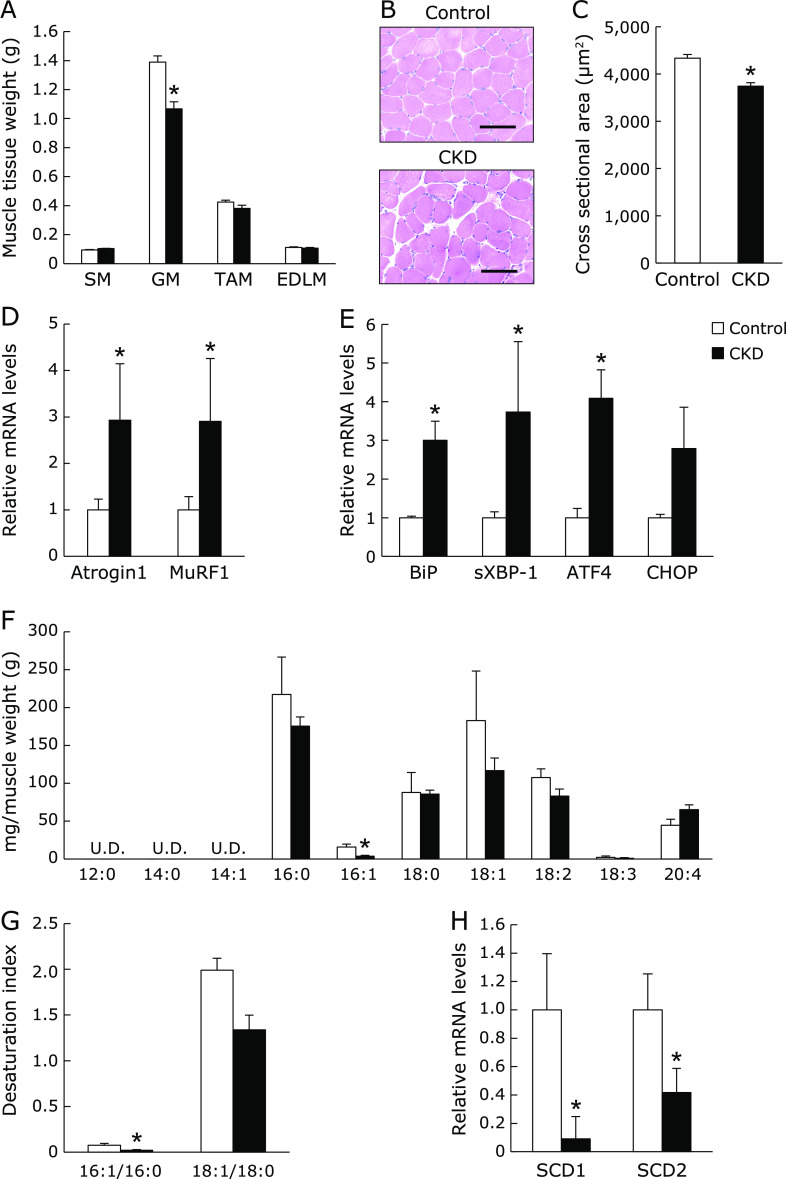
The reduction of *SCD1* and *SCD2* mRNA expression in gastrocnemius muscle of adenine-induced CKD model rats. Eight-week-old male Wistar rats were fed either 0.3% adenine containing diet (CKD) or control diet (Control) for 6 weeks. (A) The skeletal muscle weight. SM, soleus muscle; GM, gastrocnemius muscle; TAM, tibialis anterior muscle; EDLM, extensor digitorum longus muscle. (B) Cross-sections in gastrocnemius muscle were stained with H&E. Scale bar = 100 µm. (C) Muscle fiber cross-sectinonal area (µm^2^) in gastrocnemius muscle. Mean ± SEM. (*n* = 200). (D, E, H) The muscle-specific ubiquitin E3 ligases (*Atrogin1* and *MuRF1*), the UPR (*BiP*, *sXBP-1*, *ATF4*, and *CHOP*), *SCD1*, and *SCD2* mRNA levels in gastrocnemius muscle were evaluated by real-time PCR analysis. (F, G) Fatty acids composition and desaturation index (16:1/16:0 ratio and 18:1/18:0 ratio) in gastrocnemius muscle were analyzed by gas-liquid chromatography. U.D., undetectable. Mean ± SEM. (*n* = 3–5). *p<0.05 vs control.

First, we prepared adenine-induced CKD model rats to determine whether CKD causes a change of fatty acid metabolism in skeletal muscles. Serum levels of BUN, creatinine, and phosphorus were significantly increased in CKD rats compared with control rats as previously reported.^(22)^ In contrast, CKD rats showed the decrease of serum levels of calcium and 1,25(OH)_2_D compared with control (Table 3). CKD rats also displayed a significant reduction of muscle weight in GM compared with control, but not other skeletal muscles (Fig. 1A). Likewise, H&E staining and the measurement of muscle fiber size revealed that CKD rats decreased the mean cross-sectional area of GM compared with control (Fig. 1B and C). The E3 ubiquitin ligases determine the selectivity and specificity of the UPS. In particular, muscle RING finger-1 (MuRF1) and atrogin1, which are muscle-specific E3 ubiquitin ligases, can play a key role in muscle proteolysis and atrophy.^(23)^ We performed real-time quantitative PCR analysis to measure the muscle-specific ubiquitin E3 ligase and UPR gene expression in GM. The levels of the *Atrogin1* and *MuRF1* mRNA were significantly increased in GM of CKD rats compared with control (Fig. 1D). CKD rats increased the *BiP*, *sXBP-1*, *ATF4*, and *CHOP* mRNA expression levels in GM (Fig. 1E). Next, the fatty acid composition of GM was analyzed using gas-liquid chromatography. The levels of 16:1 was significantly decreased, and the levels of 18:1 was a decreased tendency in CKD rats (Fig. 1F). In contrast, the levels of SFAs (16:0 and 18:0) and polyunsaturated fatty acids (18:2, 18:3, and 20:4) were not altered in GM of CKD rats. In addition, CKD rats decreased the desaturation index (16:1/16:0 and 18:1/18:0) in GM compared with control (Fig. 1G). Because SCD desaturases 16:0 to 16:1 and 18:0 to 18:1, we determined the *SCD1* and *SCD2* mRNA expression in GM by real-time quantitative PCR analysis. CKD rats significantly decreased the mRNA levels of *SCD1* and *SCD2* in GM compared with control rats (Fig. 1H).

### MUFA blocks SCDi-induced atrophy through the decrease of ER stress in myotubes

**Fig. 2:**
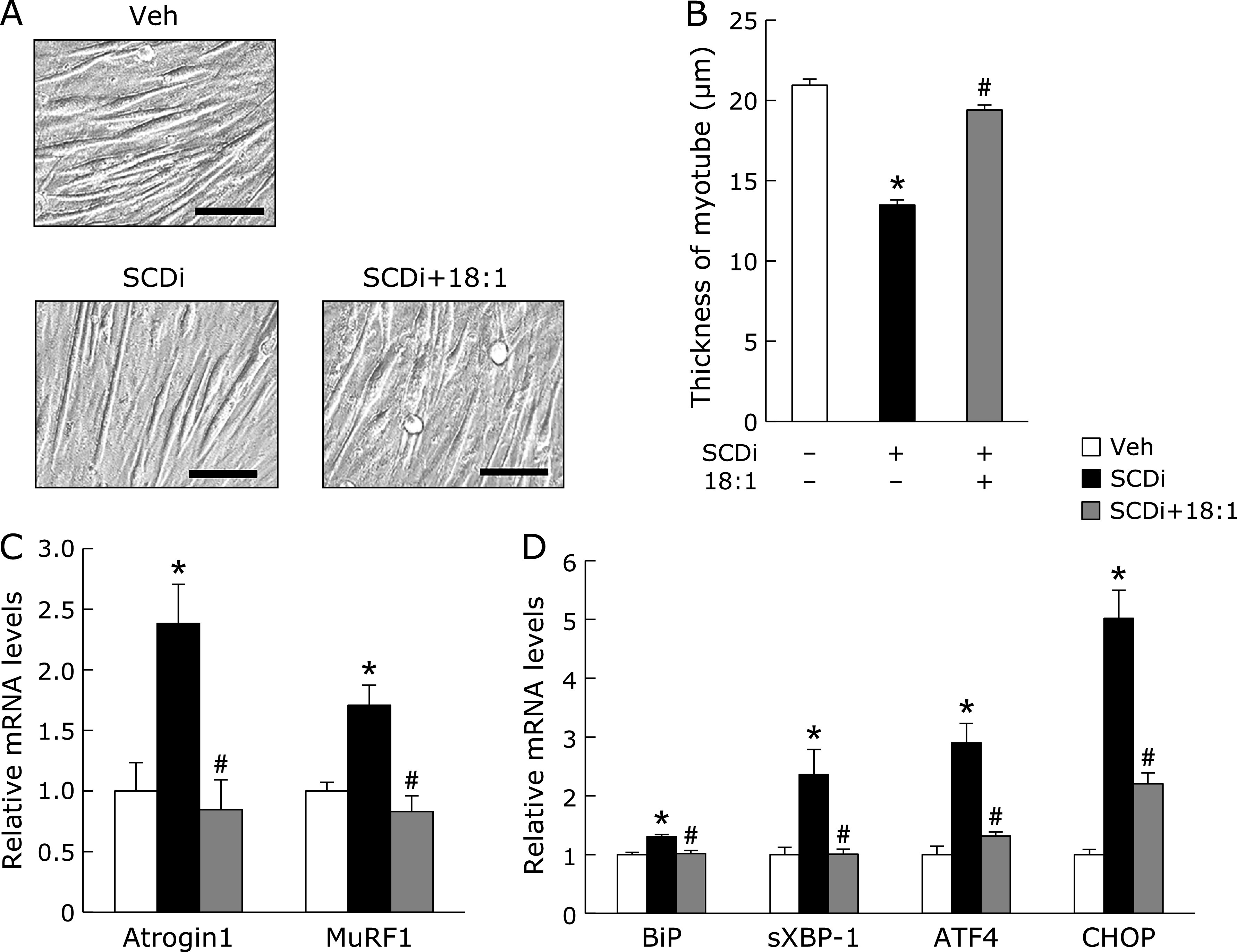
Effects of oleic acid on C2C12 myotube treated with SCD inhibitor. C2C12 cells were starved for 6 days to be differentiated into myotubes cells. Myotube cells were treated with vehicle (Veh), 1 µM SCD inhibitor (SCDi, CAY10566), and 300 µM oleic acid (18:1) for 48 h (A–C) or 12 h (D). (A) Representative images of myotubes treated as indicated. Scale bar = 100 µm. (B) The measurement of diameters as described in MATERIAL and METHODS. Mean ± SEM. (*n* = 100). (C, D) The muscle-specific ubiquitin E3 ligases (*Atrogin1* and *MuRF1*) and the UPR (*BiP*, *sXBP-1*, *ATF4*, and *CHOP*) mRNA expression were evaluated by real-time PCR analysis. Mean ± SEM. (*n* = 3). *p<0.05 vs Veh, ^#^*p*<0.05 vs SCDi.

It has been reported that 18:1 inhibits 18:0-induced ATF4 expression in vascular smooth muscle cells.^(15)^ In addition, 18:1 prevents 16:0-induced atrophy through modulation for mitochondrial reactive oxygen species production in C2C12 myotubes.^(16)^ First, we investigated whether 18:1 blocks ER stress induced by SCDi in C2C12 myotubes. As previously reported, the thickness of C2C12 myotubes differentiated by the starvation for 6 days was markedly decreased by SCDi, which was significantly increased by co-treatment with 18:1 (Fig. 2A and B). Furthermore, SCDi-induced the increase of the levels of the *Atrogin1* and *MuRF1* mRNA in C2C12 myotubes were restored to DMSO treatment by co-treatment with 18:1 (Fig. 2C). Likewise, the levels of the *BiP*, *sXBP-1*, *ATF4*, and *CHOP* mRNA expression were increased by SCDi in C2C12 myotubes, which were significantly decreased by co-treatment with 18:1 (Fig. 2D).

**Fig. 3:**
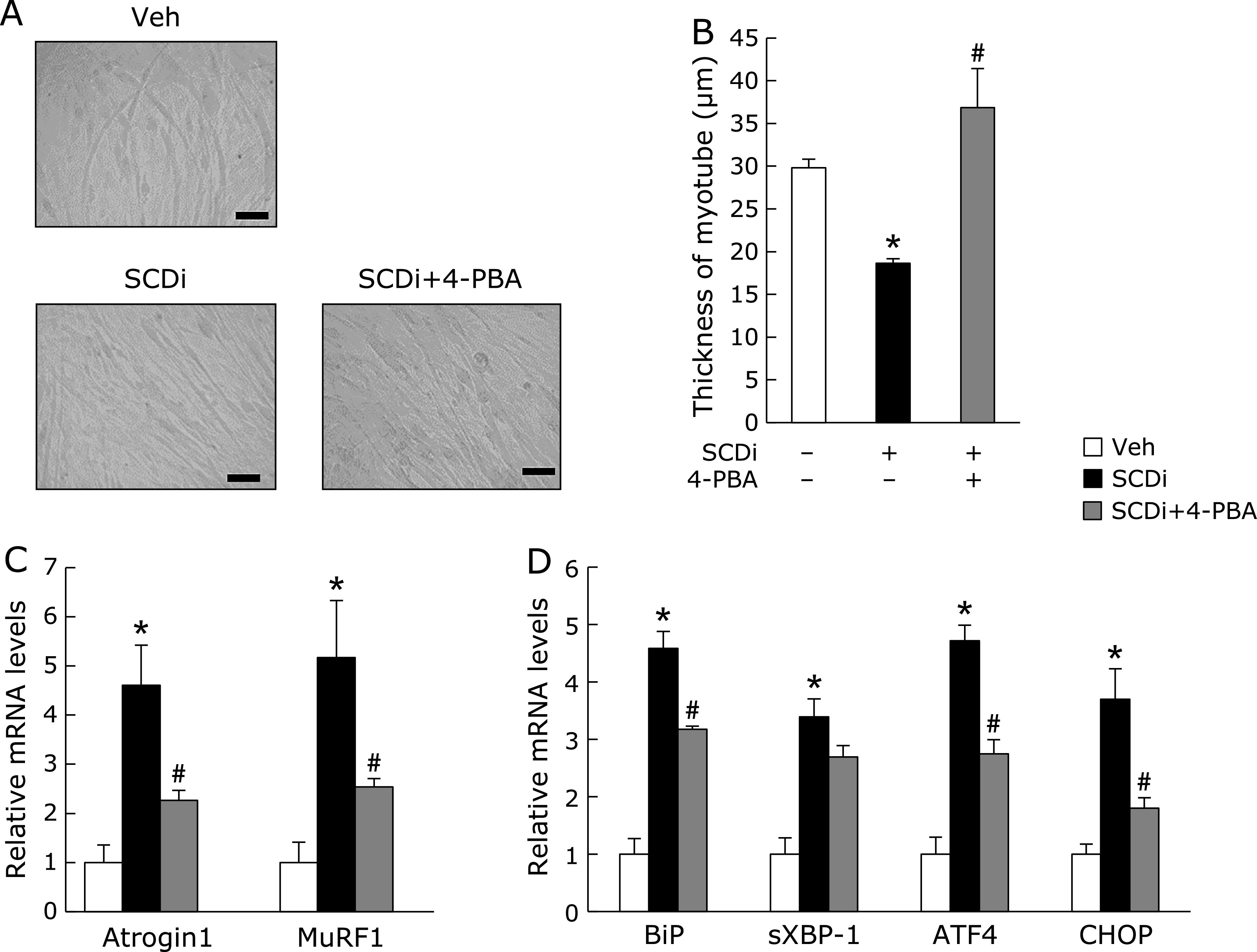
Effects of chemical chaperone on C2C12 myotube treated with SCD inhibitor. C2C12 cells were starved for 6 days to be differentiated into myotube cells. Myotube cells were treated with vehicle (Veh), 1 µM SCD inhibitor (SCDi, CAY10566) and 5 mM 4-PBA for 48 h (A–C) or 12 h (D). (A) Representative images of myotubes treated as indicated. Scale bar = 100 µm. (B) The measurement of diameters as described in MATERIAL and METHODS. Mean ± SEM. (*n* = 100). (C, D) The muscle-specific ubiquitin E3 ligases (*Atrogin1* and *MuRF1*) and the UPR (*BiP*, *sXBP-1*, *ATF4*, and *CHOP*) mRNA expression were evaluated by real-time PCR analysis. Mean ± SEM. (*n* = 3). *p<0.05 vs Veh, ^#^*p*<0.05 vs SCDi.

Next, since it has been reported that ATF4 is a critical mediator of atrophy in aged skeletal muscle,^(7)^ we investigated whether chemical chaperone, such as 4-PBA which is known to attenuate ER stress, normalized atrophy induced by SCDi in C2C12 myotubes. The thickness of C2C12 myotubes differentiated by the starvation for 6 days was markedly decreased by SCDi, which was significantly increased by co-treatment with 4-PBA (Fig. 3A and B). In C2C12 myotubes treated with SCDi, 4-PBA significantly reduced the levels of *Atrogin1* and *MuRF1* mRNA expression (Fig. 3C). Furthermore, we confirmed 4-PBA greatly diminished the levels of the *BiP*, *sXBP-1*, *ATF4*, and *CHOP* mRNA expression increased by SCDi in C2C12 myotubes (Fig. 3D). These results demonstrated that the increase of SFAs and the decrease of MUFAs induced by SCDi can be involved myotube atrophy via ER stress.

### Chemical chaperon improved muscle atrophy in CKD model mice

**Fig. 4:**
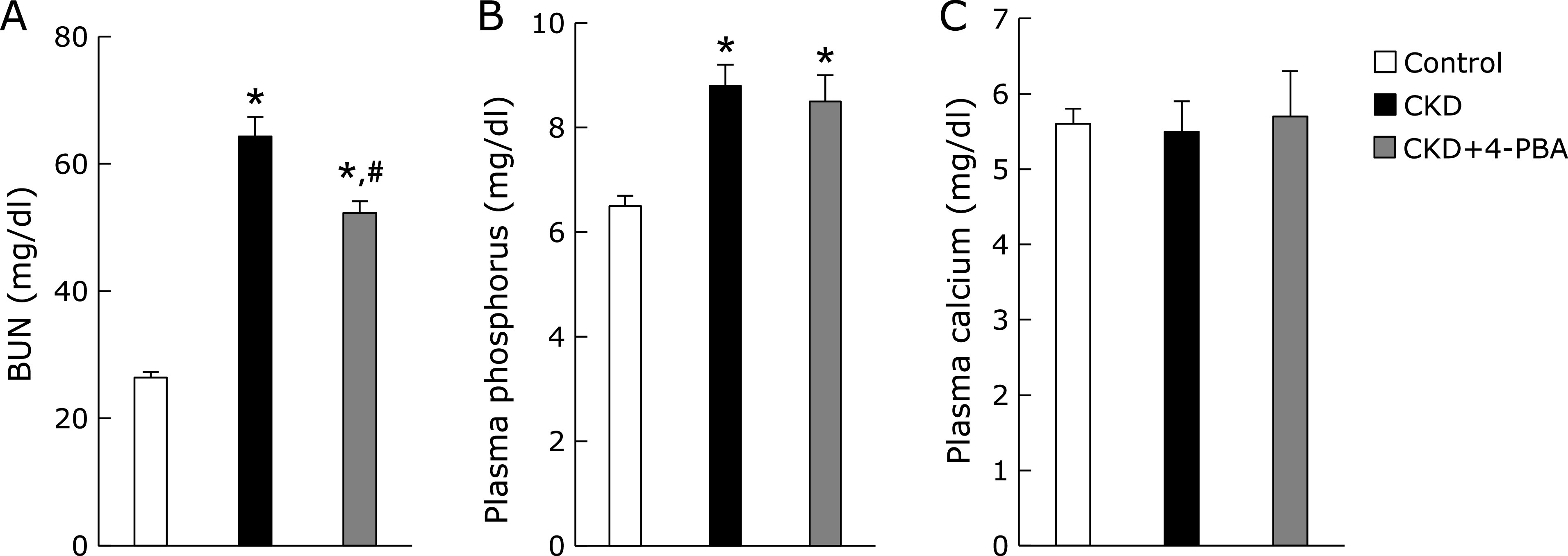
Effects of chemical chaperone on biochemical data in adenine-induced CKD model mice. Eight-week-old male C57BL/6J mice were fed either 0.2% adenine containing diet with or without chemical chaperone (4-PBA), or control diet for 6 weeks. (A) Blood urea nitrogen (BUN) concentrations. (B) Plasma phosphorus concentrations. (C) Plasma calcium concentrations. Mean ± SEM. (*n* = 4–7). *p<0.05 vs control, ^#^*p*<0.05 vs CKD.

**Fig. 5:**
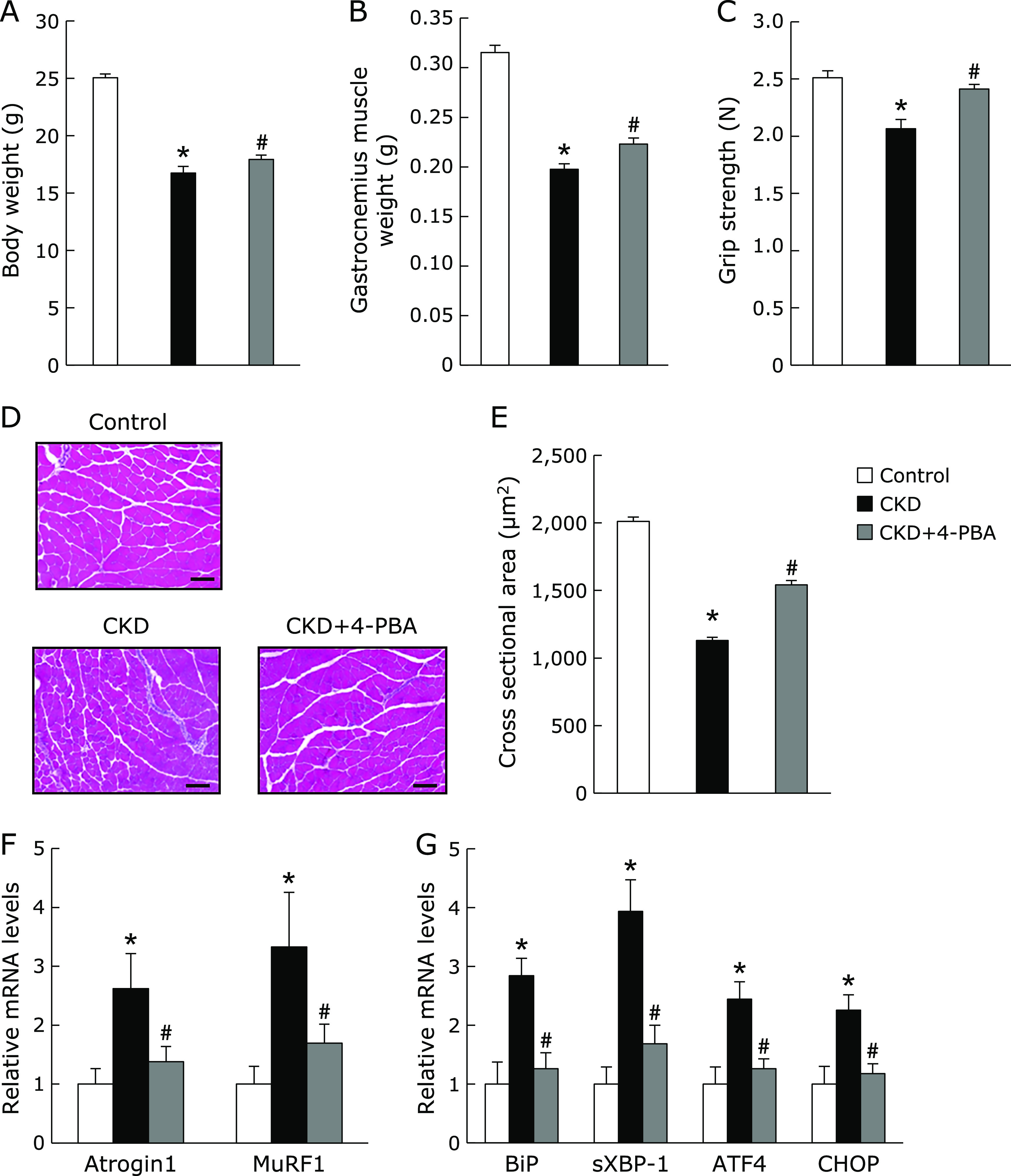
Effects of chemical chaperone on muscle atrophy in adenine-induced CKD model mice. Eight-week-old male C57BL/6J mice were fed either 0.2% adenine containing diet with or without chemical chaperone (4-PBA), or control diet for 6 weeks. (A) Body weight. (B) The gastrocnemius muscle weight. (C) Grip strength. (D) Cross-sections in gastrocnemius muscle were stained with H&E. Scale bar = 100 µm. (E) Muscle fiber cross-sectinonal area (µm^2^) in gastrocnemius muscle. Mean ± SEM (*n* = 100). (F) The muscle-specific ubiquitin E3 ligases (*Atrogin1* and *MuRF1*) and the UPR mRNA expressions (*BiP*, *sXBP-1*, *ATF4*, and *CHOP*) mRNA levels in gastrocnemius muscle were evaluated by real-time PCR analysis. Mean ± SEM. (*n* = 4–7). *p<0.05 vs control, ^#^*p*<0.05 vs CKD.

Recently, some groups have reported that the inhibition of ER stress by chemical chaperone attenuated the progression of CKD model animals.^(24,25)^ To determine the effects of 4-PBA on muscle atrophy in CKD we investigated adenine-induced CKD model mice treated with 4-PBA or vehicle. As expected, the increase of the levels of BUN in CKD mice was significantly inhibited by treatment with 4-PBA (Fig. 4A). Levels of plasma phosphorus were increased in CKD mice, which was not affected by 4-PBA treatment (Fig. 4B). Plasma calcium levels did not differ among all groups (Fig. 4C). Treatment of 4-PBA significantly ameliorated the reduction of the body and GM weight in CKD mice (Fig. 5A and B). In addition, the weakness of grip strength in CKD mice was significantly improved by 4-PBA (Fig. 5C). Similarly, H&E staining and the measurement of muscle fiber size revealed that treatment of 4-PBA significantly improved the mean cross-sectional area in GM of CKD mice (Fig. 5D and E). Real-time quantitative PCR analysis demonstrated that 4-PBA significantly reduced the levels of gene expression of the muscle-specific ubiquitin E3 ligase and UPR in GM of CKD mice (Fig. 5F and G).

## Discussion

Skeletal muscle atrophy, which is a complication of CKD, contributes to mortality in CKD patients.^(2)^ However, the underlying mechanism by which CKD causes muscle atrophy has not been completely elucidated. In this study, we concluded that the excessive ER stress through the repression of SCD activity in skeletal muscle causes muscle atrophy in CKD. SCD is an ER transmembrane enzyme that regulates the level of SFAs in the ER by converting 16:0 to 16:1 and 18:0 to 18:1, respectively. In the past, we previously reported that CKD mice had lower levels of the *SCD1* and *SCD2* mRNA expression and the activity of SCD in the aortic medial layers compared with control. Also, patients with stage 3 and 4 CKD had higher levels of serum stearic acid but not oleic acid, as well as a consequent lower desaturation index that is reflective of SCD activity compared with subjects with normal kidney function.^(15)^ In the present study, we confirmed that the amount of 16:1, the desaturation index (16:1/16:0), and the levels of *SCD* mRNA expression were significantly decreased in GM of CKD rats compared with control. On the other hand, it is well-known that CKD patients show hyperphosphatemia.^(1)^ We have recently indicated that elevation of extracellular phosphorus level reduces SCD activity and its protein and mRNA levels in human vascular smooth muscle cells, and rats fed with high phosphorus diets decline the levels of *SCD* mRNA expression in white adipose tissue compared with control diets.^(26)^ In fact, high phosphorus loading in C2C12 myotubes decreased the levels of mRNA expression**of *SCD1* (data not shown). Together, these results suggest that hyperphosphatemia in CKD rats may be involved in the reduction of the SCD mRNA expression and its activity in GM.

Lipoquality has recently been a focus of study in the pathology of metabolic diseases. Some groups suggest that SFAs and MUFAs have distinct effects on the peripheral organs by each lipotoxicity, which is defined as the ectopic accumulation of lipid intermediate and final products. Previously, we have reported that the increase of SFAs (16:0 and 18:0) by the reduction of SCD activity in the medial layer of aortas of CKD mice mediates vascular calcification.^(15)^ It has also been reported that 18:1 prevents 16:0-induced atrophy in C2C12 myotubes.^(17)^ In the present study, the increase of SFAs by SCDi in C2C12 myotubes induced muscle atrophy and the excess of ER stress, which were blocked by the co-treatment with 18:1. Since it has been suggested that excessive ER stress is associated with the pathogenesis of muscle atrophy,^(6,7)^ we investigated the effects of the depression of ER stress by a chemical chaperone, 4-PBA, on the thickness of C2C12 myotubes and muscle atrophy in GM of CKD mice. As we expected, we demonstrated that the attenuation of ER stress by 4-PBA resulted in a significant recovery of the reduction of the thickness of C2C12 myotubes induced by SCDi. Furthermore, 4-PBA treatment ameliorated the loss of the GM weight, the weakness of grip strength, and the decrease of the mean cross-sectional area in GM of CKD mice. Taken together, these results suggest that excess ER stress induced by the decreased ratio of MUFAs/SFAs in skeletal muscle may contribute to CKD-induced muscle atrophy. However, previous studies have indicated that the inhibition of ER stress by a chemical chaperone inhibits the progression of CKD in model animals.^(24,25)^ In this study, 4-PBA decreased the levels of BUN in CKD mice. Therefore, the ameliorating effects of 4-PBA on the CKD-induced muscle atrophy in GM might be partially through the improvement of renal function.

Interestingly, docosahexaenoic acid (DHA: 22:6), which is polyunsaturated fatty acids (PUFAs), prevents 16:0-induced activation of caspase-mediated proteolysis, in part, by preventing excessive ER stress.^(16)^ Our previous results have also shown that co-treatment with linoleic acid (18:2) and 22:6 completely blocks mineralization and ER stress of vascular smooth muscle cells induced by SCDi.^(15)^ The fatty acid composition of phospholipids, which is an important component of biological membranes, often shows clear correlations with the composition of dietary fats.^(27,28)^ The fatty acid species in the phospholipid bilayer greatly influence its properties. A high SFA content increases membrane rigidity, while a high MUFA/PUFA content promotes fluidity.^(29)^ Interestingly, reduced ER membrane fluidity has the potential of mediating ER stress through the regulation of ER calcium channels.^(30)^ Although we have not investigated ER membrane fluidity in the present study, the reduction of SCD activity may cause ER stress via reducing ER membrane fluidity in skeletal muscle of CKD animal and in C2C12 myotubes.

Forkhead transcription factors (FoxOs) cause skeletal muscle atrophy through the transcriptional regulation of the *Atrogin1* gene promoter.^(31)^ Interestingly, Okamura *et al.*^(32)^ reported that the sodium glucose cotranspoter-2 inhibitor (SGLUT-2i) results in the suppression of both the increased *FoxO1* expression and the reduced muscle cross-sectional area in SM of genetically diabetic *db/db* mice. This report indicated that SGLUT-2i suppresses muscle atrophy in *db/db* mice by reducing the expression of FoxO1. In addition, FoxO represses the expression of SREBP1c which upregulates the SCD expression.^(33)^ From our results, SGLUT-2i may inhibit muscle atrophy in *db/db* mice through the reduced FoxO1 which not only directly but also indirectly affects the Atrogin1 expression by the SCD expression.

**Fig. 6:**
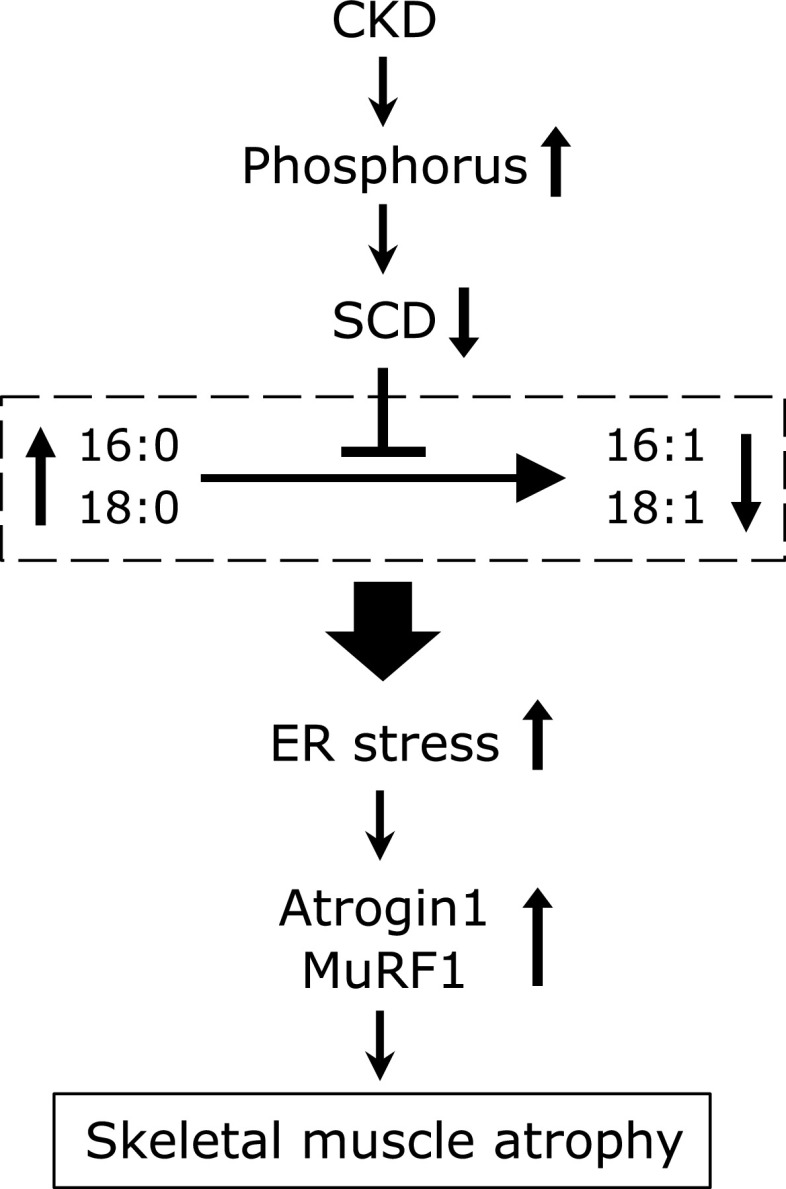
Schematic diagram of CKD-induced muscle atrophy through a reduction of SCD activity. Reduced SCD activity leads to an increase in SFAs (16:0 and 18:0) levels and a decrease in MUFAs (16:1 and 18:1) in skeletal muscle of CKD. A decrease in the ratio of MUFAs/SFAs causes ER stress, resulting in muscle atrophy by the induction of muscle-specific E3 ubiquitin ligases (Atrogin1 and MuRF1).

In conclusion, our findings reveal that the excess ER stress induced by a decrease in the ratio of MUFAs/SFAs in skeletal muscle contribute CKD-induced muscle atrophy. As indicated in Fig. 6, we propose that CKD reduce SCD expression by increased phosphorus. Reduced SCD activity leads to an increase in SFAs levels and a decrease in MUFAs in skeletal muscle. A decrease in the ratio of MUFAs/SFAs causes ER stress, resulting in muscle atrophy through the induction of muscle-specific E3 ubiquitin ligases such as Atrogin1 and MuRF1.

## Figures and Tables

**Table 1:** The compositions of the experimental diet

	Control diet (rat and mouse)	rat CKD diet (Adenine 0.3%)	mouse CKD diet (Adenine 0.2%)	mouse CKD + 4-PBA diet (Adenine 0.2%)
Altered AIN-93G	59.5	59.5	59.5	59.5
Milk casein	20	20	20	20
Sugar	4.77	4.77	4.77	4.77
Mineral Mix	1.56	1.56	1.56	1.56
CaCO_3_	1.26	1.26	1.26	1.26
Soy bean oil	7	7	7	7
KH_2_PO_4_	3.49	3.49	3.49	3.49
KCl	0.48	0.48	0.48	0.48
Dextrin	1.94	1.64	1.74	0.86

Adenine	0	0.3	0.2	0.2

4-PBA	0	0	0	0.88

Total (g)	100	100	100	100

**Table 2:** Sequence of oligonucleotide primers for real-time quantitative PCR analysis

Gene name	Forword	Reverse
rat SCD1	5'-CTACAAGCCTGGCCTCCTGC-3'	5'-GGACCCCAGGGAAACCAGGA-3'
rat SCD2	5'-TGCACCCCCAGACACTTGTAA-3'	5'-GGATGCATGGAAACGCCATA-3'
rat Atrogin1	5'-CCATCAGGAGAAGTGGATCTATGTT-3'	5'-GCTTCCCCCAAAGTGCAGTA-3'
rat MuRF1	5'-GTGAAGTTGCCCCCTTACAA-3'	5'-TGGAGATGCAATTGCTCAGT-3'
rat BiP	5'-CCTGTTCTGGACTCTGTGA-3'	5'-AGGAGTGAAGGCCACATACG-3'
rat sXBP-1	5'-GCTTGTGATTGAGAACCAGG-3'	5'-GGCCTGCACCTGCTGCGGACTC-3'
rat ATF4	5'-ACCATGGCGTATTAGAGGCAG-3'	5'-TGTCCGTTACAGCAACGCT-3'
rat CHOP	5'-TGTTGAAGATGAGCGGGTGG-3'	5'-GACTCAGCTGCCATGACTGT-3'
rat β-actin	5'-GCAGGAGTACGATGAGTCCG-3'	5'-GGGTGTAAAACGCAGCTCAG-3'
mouse SCD1	5'-CCAAGCTGGAGTACGTCTGG-3'	5'-CAGAGCGCTGGTCATGTAGT-3'
mouse SCD2	5'-GCATTTGGGAGCCTTGTACG-3'	5'-AGCCGTGCCTTGTATGTTCTG-3'
mouse Atrogin1	5'-TCCAGACCCTCTACACATCCTT-3'	5'-CCTCTGCATGATGTTCAGTTGT-3'
mouse MuRF1	5'-GAGGGCCATTGACTTTGGGA-3'	5'-TTTACCCTCTGTGGTCACGC-3'
mouse BiP	5'-TCAGCATCAAGCAAGGATTG-3'	5'-GCTTCATGGTAGAGCGGAAC-3'
mouse sXBP-1	5'-GAGTCCGCAGCAGGTG-3'	5'-GTGTCAGAGTCCATGGGA-3'
mouse ATF4	5'-GAGCTTCCTGAACAGCGAAGTG-3'	5'-TGGCCACCTCCAGATAGTCATC-3'
mouse CHOP	5'-GAACCTGAGGAGAGAGTGTTCC-3'	5'-CAAGGTGAAAGGCAGGGACTC-3'
mouse 18S	5'-ACGGAAGGGCACCACCAGGA-3'	5'-CACCACCACCCACGGAATCG-3'

**Table 3:** Biochemical data in adenine-induced CKD model rats

	Control	CKD
BUN (mg/dl)	19.6 ± 0.8	218.5 ± 25.8*
Serum creatinine (mg/ml)	0.4 ± 0.1	3.0 ± 0.1*
Serum phosphorus (mg/dl)	6.2 ± 0.6	11.1 ± 1.1*
Serum calcium (mg/dl)	7.9 ± 0.1	7.0 ± 0.5*
Serum 1,25(OH)_2_D (pg/ml)	33.7 ± 6.5	6.1 ± 0.7*

Eight-week-old male Wistar rats were fed either 0.3% adenine diets (CKD) or vehicle (control) for 6 weeks. Mean ± SEM (*n* = 4–7). *p<0.05 vs control.
